# Annealing-free fabrication of high-quality indium tin oxide films for free-carrier-based hybrid metal–semiconductor nanophotonics

**DOI:** 10.1038/s41598-023-45651-w

**Published:** 2023-10-28

**Authors:** Alexander Korneluk, Julia Szymczak, Tomasz Stefaniuk

**Affiliations:** Faculty of Physics, University of Warsaw, Pasteura 5 St., 02-093 Warsaw, Poland

**Keywords:** Materials for optics, Metamaterials, Nanophotonics and plasmonics, Nonlinear optics, Ultrafast photonics, Electronic devices, Materials science, Optics and photonics, Optical materials and structures

## Abstract

Recent discoveries have revealed that indium tin oxide (ITO), due to the presence of an epsilon-near-zero (ENZ) point and suitable carrier concentration and mobility, can be used to modulate the refractive index, confine fields in the nanoscale, enhance nonlinear effects, achieve ultrafast light switching or to construct so-called time-varying media. While this potential positions ITO as a key material for future nanophotonic devices, producing ITO films with precisely engineered properties remains a significant challenge. Especially when the device’s complex geometry or incorporated materials require the fabrication process to be conducted at substrate temperatures below 100 °C and without any post-annealing treatment. Here we present a comprehensive study on the low-temperature deposition of 70 nm thick ITO films using an e-beam PVD system. The nanolayers evaporated under different conditions were characterized by SEM and AFM microscopy, Hall effect measurement system as well as spectroscopic ellipsometry. We discuss the factors influencing the optical, electrical, and morphological properties of ITO films. We show that smooth nanolayers of similar quality to annealed samples can be obtained at 80 °C by controlling the oxygen plasma parameters, and the ENZ wavelength can be tuned throughout the NIR spectral range. Finally, we show that using the proposed methodology, we fabricated ITO films with resistivity as low as 5.2 × 10^–4^ Ω cm, smooth surface with RMS < 1 nm, high carrier concentration reaching 1.2 × 10^21^ cm^−3^ and high transmittance (85%) in the Vis/NIR spectrum.

## Introduction

Transparent conductive oxides (TCOs) and their best-known representative—indium tin oxide (ITO), have been extensively used in various optoelectronic applications in both research and industry over the past few decades. ITO is a n-type wide-bandgap semiconductor and has emerged as one of the most popular TCOs due to its high optical transmittance in the visible and near-infrared regions along with its excellent electrical conductivity^1, 2^. It is a widely used material in everyday life devices ranging from solar cells and touch panels to organic light-emitting diodes^3–5^. In recent years, however, ITO and other TCOs have been rediscovered by the scientific community and are once again brought at the forefront of research. This is because in TCOs, carrier concentration and mobility levels can be adjusted in depth through doping and modifying deposition conditions. As a result, new fascinating and technologically important optical effects associated with free electrons in the material can be brought out. The ability to directly control the properties of the electron gas opened up new promises. Firstly, it is possible to engineer the epsilon-near-zero (ENZ) wavelength in TCOs, i.e., the wavelength at which the real part of the permittivity crosses zero. ENZ materials exhibit various exciting properties and can be used for field localization^6^, nonlinear effect enhancement^7^, dispersion management^8^, phase-matching relaxation^9^, ultrafast switching^10^, frequency shifting^11, 12^, polarization switching^13^, high-harmonic generation^14^ or metatronic devices^15^. Secondly, the carrier characteristics can be optimized in such a way that the optical losses in the system are reduced. Thus, TCOs have been perceived as alternative materials for plasmonic-based devices, offering an advantage over traditional noble metals^4, 16^. Finally, it has become feasible to electrostatically tune the local charge carrier concentration by applying external voltage. This is viable in configurations where the TCO layer is composed in a metal–oxide–semiconductor (MOS) geometry and has the proper initial carrier density. Such an active photonic structure that utilizes ITO was first proposed by Feigenbaum et al.^17^ and exhibited the unique property of a unity-order local refractive index change as an effect of voltage-induced formation of the carrier accumulation layer. This concept has paved the way for various designs of tunable devices, being absorbers^18–20^, plasmonic nanoantennas^21^, metasurfaces^22^ or silicon-based modulators^23, 24^ to name a few.

The abovementioned potential of TCOs, and in particular ITO, to serve as key materials for future nanophotonic devices depends heavily on the ability to fabricate semiconductor layers with precisely engineered electrical and optical properties. Over the years, various techniques have been utilized to fabricate ITO films, including e-beam physical vapor deposition (e-PVD)^25, 26^, sputtering^27^, sol–gel process^28^, chemical vapor deposition^29^, or pulsed laser deposition^30^. The established position of ITO in modern, everyday optoelectronic devices might suggest that the manufacturing processes for ITO films are well known. However, the fabrication can become complicated when specific details are considered. Two aspects are worth discussing. Firstly, the quality of ITO is dependent on a number of process conditions, with the substrate temperature during the deposition phase being the most critical factor according to reported studies. To obtain high carrier concentration and optical transparency simultaneously, it is necessary to introduce high substrate temperatures, typically between 250 and 500 °C, to ensure sufficient recrystallization of the evaporated material^2, 25, 26, 30–33^. As an alternative, it is also suggested to use post-process annealing at 550 °C to produce samples of similar quality^15, 16, 34, 35^. Unfortunately, the introduction of elevated temperatures into the manufacturing process can damage other, more temperature-sensitive nanolayers or even entire components of the device in the case of many systems. For example, in the fabrication of flexible electronic devices or next-generation photovoltaics, which designs involve polymers, such conditions are unacceptable, as they cause thermal damage to the materials^36, 37^. The temperature constraints also limit the development of hybrid metal–semiconductor time-varying media, which could leverage the distinct characteristics of free electrons in both materials^38, 39^. The problem of having to use high temperature to fabricate high-quality ITO films has been identified in the literature, and there have been several attempts to resolve it. The research was focused on the optimization of the process conditions in either magnetron sputtering^40, 41^, pulsed layer deposition or ion-assisted e-beam deposition^42, 43^. Nevertheless, the fabricated samples still showed reduced electrical conductivity compared to those obtained using substrate heating. Furthermore, the satisfactory result was only achieved for layers thicker than 200 nm, which might exceed the desired thickness for certain nanophotonic applications. This represents the second area of concern in ITO fabrication. Specifically, it relates to the rapid degradation of the electrical properties of ITO with decreasing film thickness^30, 44^. The effect is associated with the kinetics of the initial stages of nucleation and growth of the ITO layer and the resulting general morphology of the thin film, the presence of percolation, and the size of the individual grains^30^. This is a crucial factor because not only does the structure of the layer affect the value of the optical constants, but in more complex multilayer geometries, smooth interfaces between different materials are essential to avoid scattering effects that influence the overall performance of the device.

In this work, we address the above issues and present comprehensive studies on sub-100 °C ion-assisted electron beam deposition of 70 nm thick ITO films with excellent electrical conductivity (resistivity of 5.2 × 10^–4^ Ω cm), high carrier concentration reaching 1.2 × 10^21^ cm^−3^, high transparency (85%) and low surface roughness (RMS < 1 nm). Our study is focused on the measurements of electrical and optical properties of the layer, as well as its surface morphology—a crucial factor in nanophotonic design. We provide other researchers with useful guidelines for the selection of e-beam evaporation process parameters such as deposition rate, substrate temperature, presence of oxygen gas and oxygen plasma. In particular, we show that the parameters of the oxygen plasma, namely the gas flow rate, the discharge voltage, and the discharge current, significantly affect the ITO layer properties. Finally, we demonstrate how the carrier concentration and mobility in the ITO film can be tuned to adjust the ENZ wavelength throughout the NIR spectral range.

## Electrical and morphological properties of ITO films

ITO electrical properties arise from its complex crystal structure. A unit cell of ITO resembles that of indium oxide (In_2_O_3_), containing 80 atoms, and the indium cations are located in two different six-fold-coordinated sites^2^. In this degenerated semiconductor, the majority carriers are electrons that originate from the partial substitution of indium In^3+^ by tin Sn^4+^ ions and from the creation of doubly charged oxygen vacancies O^2-^ in the lattice^2, 45^. These two mechanisms are strongly correlated with the substrate temperature used in the manufacturing process and the introduction of post-annealing treatment^35, 46^. Since ITO layers are typically prepared using physical vapor deposition techniques, the deposited films are polycrystalline and have significantly more complex carrier transport mechanisms than those found in single crystals. In such samples, the dominant electron scattering processes are associated with ionized impurities, grain barriers, and crystallographic defects, all of which limit electron mobility in thinner films surfaces and interfaces particularly, and thus increase the resistivity with accordance to the formula^46, 47^:

1 $$\rho =\frac{1}{eN\mu },$$

where $\rho$ is resistivity, $N$ electron concentration, $e$ electron charge and $\mu$ mobility. When estimating electrical properties by different methods, such as Hall effect measurements and ellipsometry, it is also important to consider the discrepancy between optical and electrical thickness. On the basis of the series of samples with ITO film thicknesses ranging from 30 to 100 nm, we have determined this difference to be 13.8 nm, which is similar to the values stated in other works^30, 48^.

**Figure 1:**
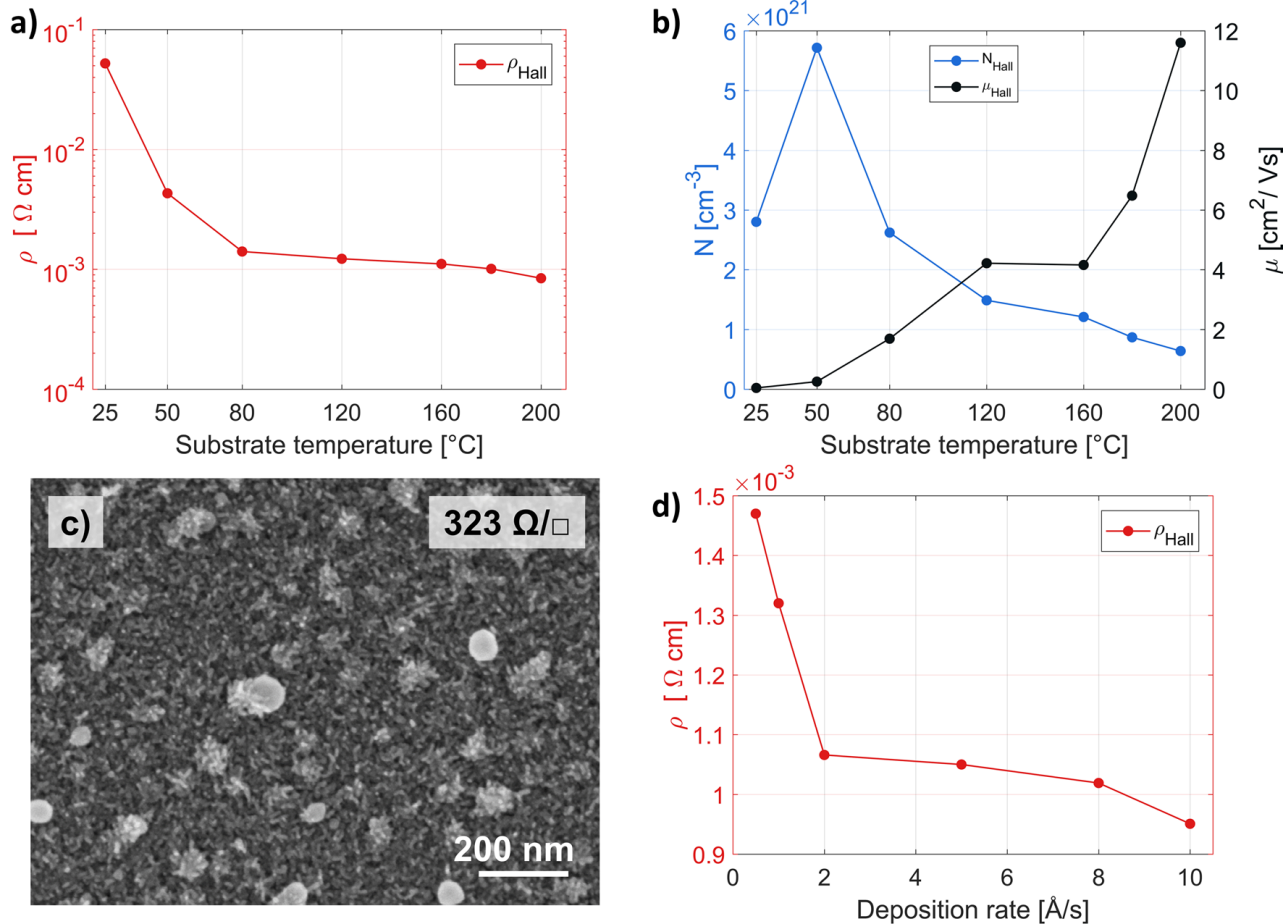
Influence of temperature on electrical properties of 70 nm thick ITO films, deposited at 1 Å/s. (**a**) Resistivity. (**b**) Hall carrier concentration and mobility. (**c**) SEM image of morphology of the sample evaporated in oxygen-free vacuum. (**d**) Resistivity of 70 nm thick ITO films as a function of deposition rate. The substrate was heated to a temperature of 80 °C.

As reported in the literature, different levels of ITO crystallinity can be achieved by selecting the PVD technique and controlling the deposition conditions. This results in films with different electrical and optical properties. Identifying the most favorable conditions for producing high-quality ITO films requires multi-parameter studies with countless number of combinations. The general concept behind our current work, however, is to develop a process that would allow for substituting indium atoms with tin and creating desired levels of oxygen vacancies without the need for high temperatures during the deposition process or post-annealing. Therefore, we start our research by only roughly determining the boundary conditions for the most important process parameters that will yield ITO layers with initially satisfactory properties, and then fine-tune the process by adjusting the oxygen gas or oxygen plasma parameters. Since thermal activation is a crucial factor in the crystallization process, our optimization procedure starts with identifying the temperature threshold below which the electrical properties of the samples rapidly deteriorate. In Fig. 1a, we present the measured resistivity values of 70 nm thick ITO films deposited at 1 Å/s rate in processes with different substrate temperatures. We observed that the conductivity of the samples improved as the substrate temperature increased. Although this is an expected general trend, the rate of improvement tends to slow down after reaching 80 °C. The analysis of carrier concentration and mobility (Fig. 1b) provides some additional information regarding the mechanisms behind this relationship. The decrease in resistivity is mainly due to the increase in carrier mobility associated with the improvement in crystallinity^47^. At the same time, the opposite effect occurs, whereby the electron concentration decreases as the temperature of the substrate increases. This is a rather unexpected result, as the data reported in the literature suggest the opposite trend^26^. However, it should be kept in mind that our preliminary tests were carried out without the use of additional oxygen or oxygen plasma sources. In such low oxygen partial pressure conditions there is a tendency towards dendritic growth (see Fig. 1c), and the presence of free carriers is mainly due to doubly charged oxygen vacancies rather than Sn doping. Based on the described results, we decided that the temperature threshold should be set at the level of 80 °C, corresponding to a resistivity of 1.4 × 10^–3^ Ω cm (235 Ω/□). Such temperature is safe for the polymers and does not damage thin metallic nanolayers^49^.

Next, we determined the optimum deposition rate, assuming the substrate is heated to 80 °C. From Fig. 1d it can be seen that the films with the lowest resistivity were obtained at a deposition rate of 10 Å/s. We attribute this to two reasons. Firstly, increasing the deposition rate leads to higher kinetic energy of the atoms or molecules, which could improve the crystallization process^43^. Secondly, raising the temperature of the material in the crucible activates more Sn ions, which effectively increases the carrier concentration and improves conductivity^26^. A deposition rate of 10 Å/s was chosen for further investigation as deposition rates above this value would significantly reduce the vacuum in the chamber, deteriorating the sample quality.

**Figure 2:**
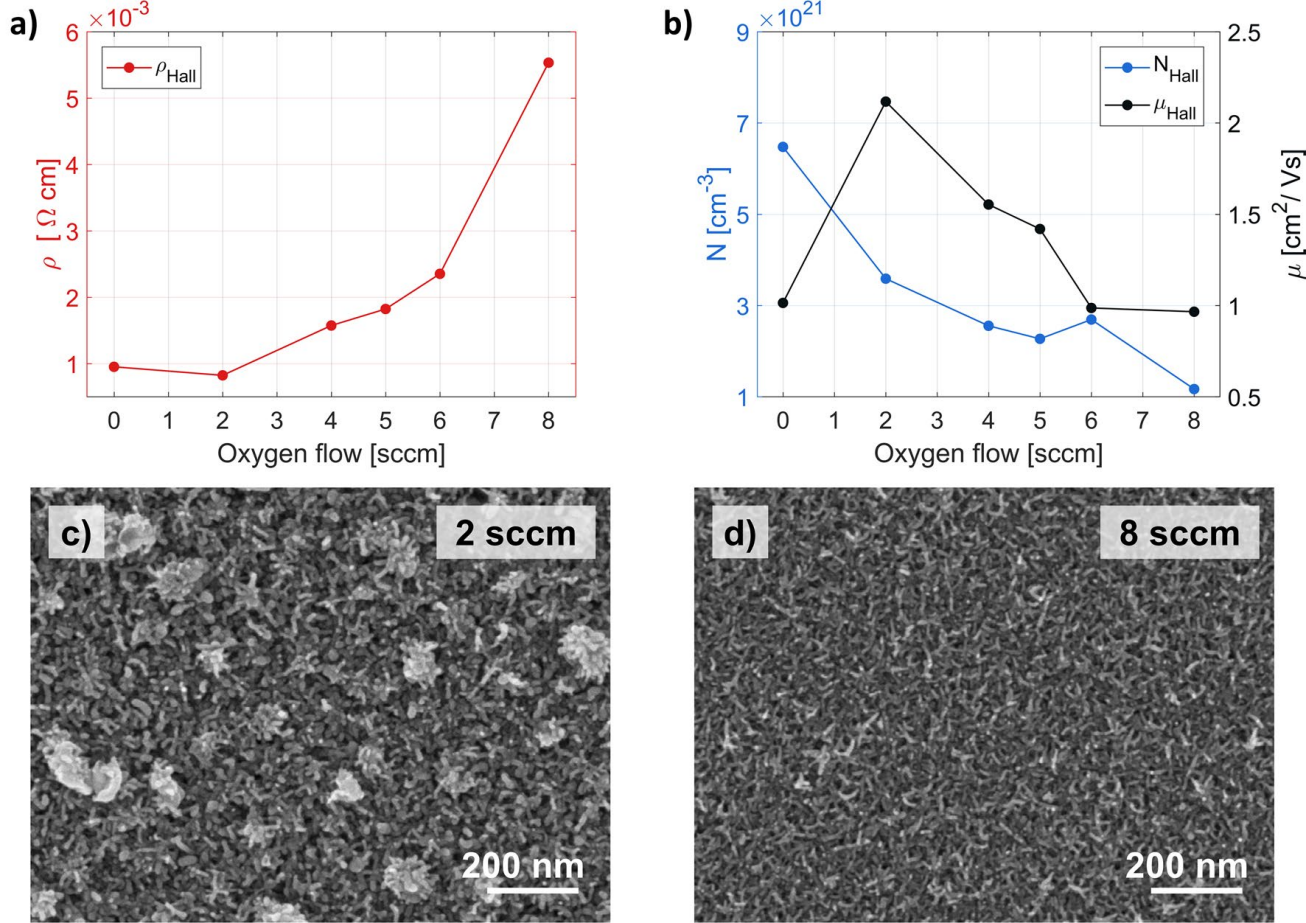
The effect of oxygen gas present during the deposition process on the electrical properties of 70 nm thick ITO films. The deposition rate was set to 10 Å/s. (**a**) Hall resistivity. (**b**) Hall carrier concentration and mobility. (**c,d**) SEM photographs of ITO films deposited with oxygen gas present in the PVD chamber. The gas flow rate is given in the upper-right corner.

In the subsequent studies, we decided to focus on the role of oxygen gas in the deposition process and its influence on the electrical properties of ITO. There are contradictory results published in the literature on this matter, with some papers suggesting a decrease in conductivity while others indicate the opposite^25, 42, 46, 50^. Therefore, we decided to make our series of customized processes with varying gas flows. In Fig. 2a,b, we present measured resistivity, carrier concentration and mobility of fabricated samples. The results indicate that as the oxygen flow rate increases, the electrical properties of the deposited films are adversely affected, and all three electrical parameters deteriorate. Several factors account for this. By incorporating oxygen atoms into ITO films, the number of oxygen vacancies, and thus, free carriers is reduced, leading to higher layer resistivity. Additional oxygen could also introduce defects and structural imperfections in the film^51^ or alter the orientation of ITO nanocrystal growth, both of which may negatively impact carrier mobility. However, we believe that in the discussed case, the most important factor influencing the electrical properties is the general surface morphology. Judging from the SEM images (Fig. 2c,d), it occurs that for the given set of parameters (deposition rate of 10 Å/s and substrate temperature of 80 °C) the neutral oxygen atoms do not participate actively in the formation of ITO structure, for any oxygen flow rate. This lack of building material forces the growing crystal to maximize its surface-to-volume ratio, leading to the dendritic structure already mentioned in the previous paragraphs^52^.

To address the insufficient quantity of oxygen atoms in the ITO structure, we decided to introduce a reactive form of oxygen into the process—oxygen plasma. We carried out two sets of depositions: (i) varying the discharge voltage, which affects the ion energy, and (ii) changing the discharge current, which influences the amount of gas converted to plasma, and thus, the number of ions reaching the sample. The oxygen flow was kept constant at 5 sccm in both series. The selected flow rate enabled us to cover a wide range of discharge currents and voltages. For the samples deposited at different discharge voltages, the discharge current was fixed at 0.4 A, whereas for the series with varying discharge current, the system was operated at a discharge voltage of 150 V. The ion bombardment was switched off immediately after the desired sample thickness was reached. In addition to the previously described results, the electrical parameters of the layers were also extracted from the ellipsometric measurements. This was possible due to a significant improvement in the optical and morphological properties in this set of samples.

**Figure 3:**
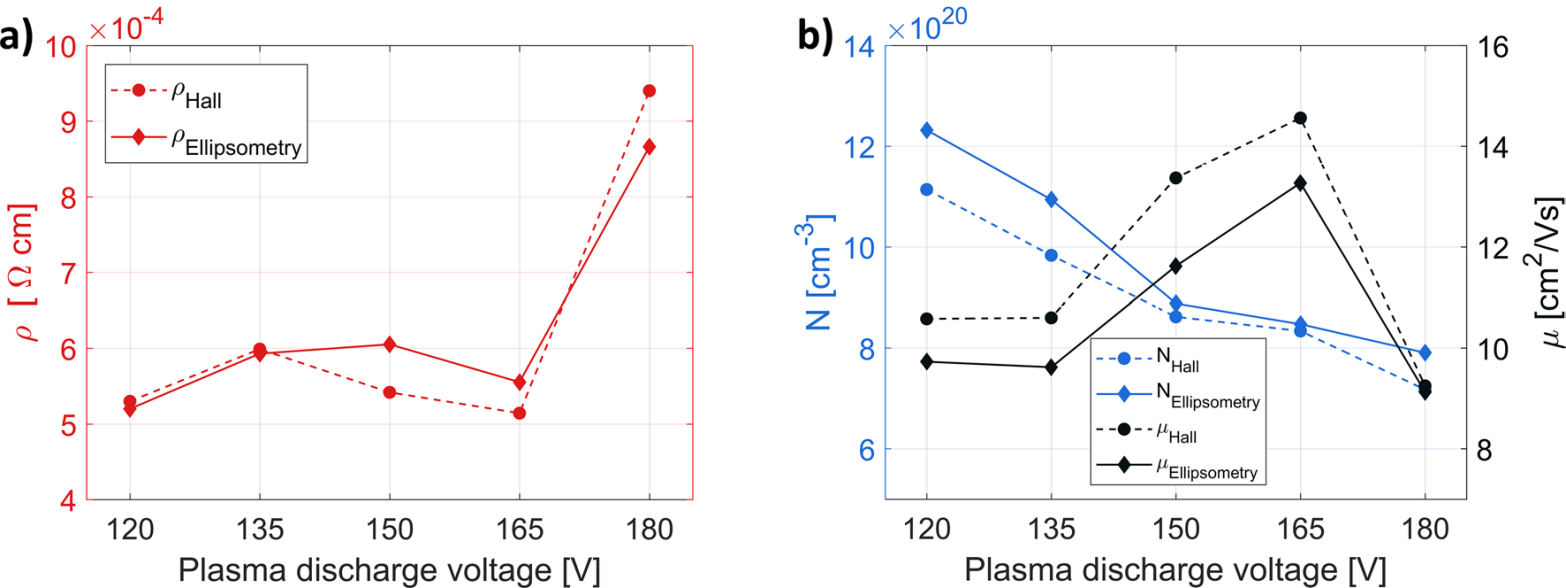
Electrical properties of 70 nm thick ITO films as a function of oxygen plasma discharge voltage, deposited at 10 Å/s, substrate temperature of 80 °C and oxygen gas flow rate 5 sccm. (**a**) Resistivity. (**b**) Hall carrier concentration and mobility. The electrical parameters derived from the ellipsometry data fitting are also marked on the corresponding figures.

**Figure 4:**
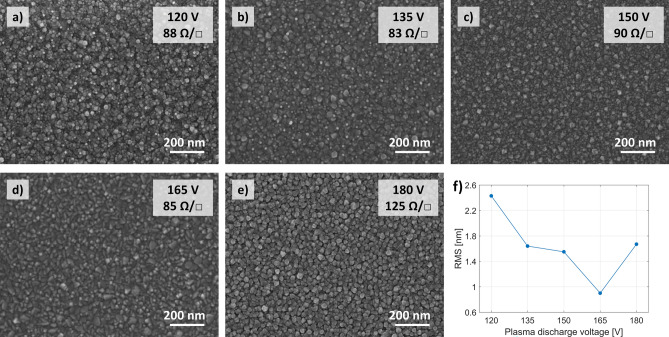
Surface morphology analysis of the ITO films deposited with oxygen ion assistance at different discharge voltages and at a constant discharge current of 0.4 A. All samples are 70 nm thick and deposited at a rate of 10 Å/s. (**a–e**) SEM photographs. The discharge voltage used for each sample and the sheet resistance measured in the Hall system are highlighted in the upper right corner. (**f**) Surface roughness RMS from AFM scans of ITO 70 nm films deposited with oxygen ions assistance as a function of the discharge voltage. The data was gathered from a 3 µm × 3 µm area for each sample.

The electrical measurement results show that the oxygen plasma has a significant impact on the conductivity of ITO layers, resulting in a reduction of the film resistance, from 1.8 × 10^–3^ Ω cm, when only pure oxygen gas was present, to less than 6.0 × 10^–4^ Ω cm for most of the discharge voltage values tested (Fig. 3a). The qualitative and quantitative agreement between the Hall and ellipsometric measurements proves the validity of the constructed ellipsometric model (see “Discussion” section). We found that increasing the discharge voltage leads to a decrease in carrier concentration and a simultaneous increase of their mobility (Fig. 3b). We attribute these changes to two main mechanisms. The first is related to the number of ionized scattering centers. The higher voltage allows for more efficient insertion of oxygen atoms into the ITO structure that reduces the number of oxygen vacancies, but also, as indicated by Yamaguchi, it leads to a drop in Sn^4+^ donor concentration^25^. In the end, there are fewer free electrons, but also fewer scattering centers. However, in layers deposited with a plasma discharge equal to 180 V, this trend breaks down because different growth conditions are favored at this voltage value. This results in visibly bigger ITO crystallites, as seen in Fig. 4e, but with some kind of structural disorder degrading the mobility. The second mechanism is related to the modification of surface morphology and the degree of crystallinity in the ITO layer. In contrast to the process with neutral oxygen, the usage of plasma has led to the formation of well-developed crystallites. As can be seen from the SEM images presented in Fig. 4, their size structure and separation between the grains depend strongly on the oxygen plasma discharge voltage, and the gradual increase in the voltage results in the increase of the grain size. This also boosts the carrier mobility in the case of some of the samples as lower density of grain boundaries reduces electron scattering. Compared to the samples deposited in the presence of neutral oxygen gas the overall mobility increased from 1–2 to 10–15 cm^2^/Vs in the series with oxygen plasma.

In addition to improving conductivity, plasma treatment also results in a smoother surface (Fig. 4f) compared to the oxygen-only deposition process. In particular, we show that 70 nm thick ITO films deposited with oxygen ion assistance can achieve surface roughness RMS as low as 0.9 nm at 165 V discharge voltage. This is a comparable result to those presented in Refs.^35, 44, 46^, given the significantly lower temperature of our process, preserved good electrical properties of ITO films and despite the smaller grain sizes in our samples. Lastly, it is worth noting that the use of oxygen plasma in the range of discharge voltages between 120 and 165 V allows for low resistivity to be maintained, while providing a very wide range of carrier concentration and mobility at the same time. This is crucial for many nanophotonic applications, as it enables low power consumption and offers a choice between high concentration or high mobility depending on the device requirements^53^.

**Figure 5:**
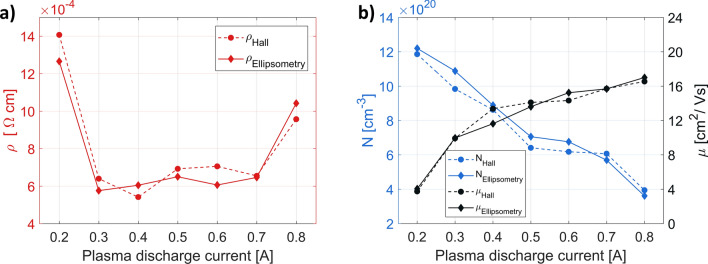
Influence of discharge current on electrical properties of 70 nm thick ITO films, deposited at 10 Å/s. The substrate temperature was 80 °C and the oxygen gas flow rate was 5 sccm. (**a**) Resistivity. (**b**) Hall carrier concentration and mobility. The electrical parameters derived from the ellipsometry data fitting are also marked on the corresponding figures.

**Figure 6:**
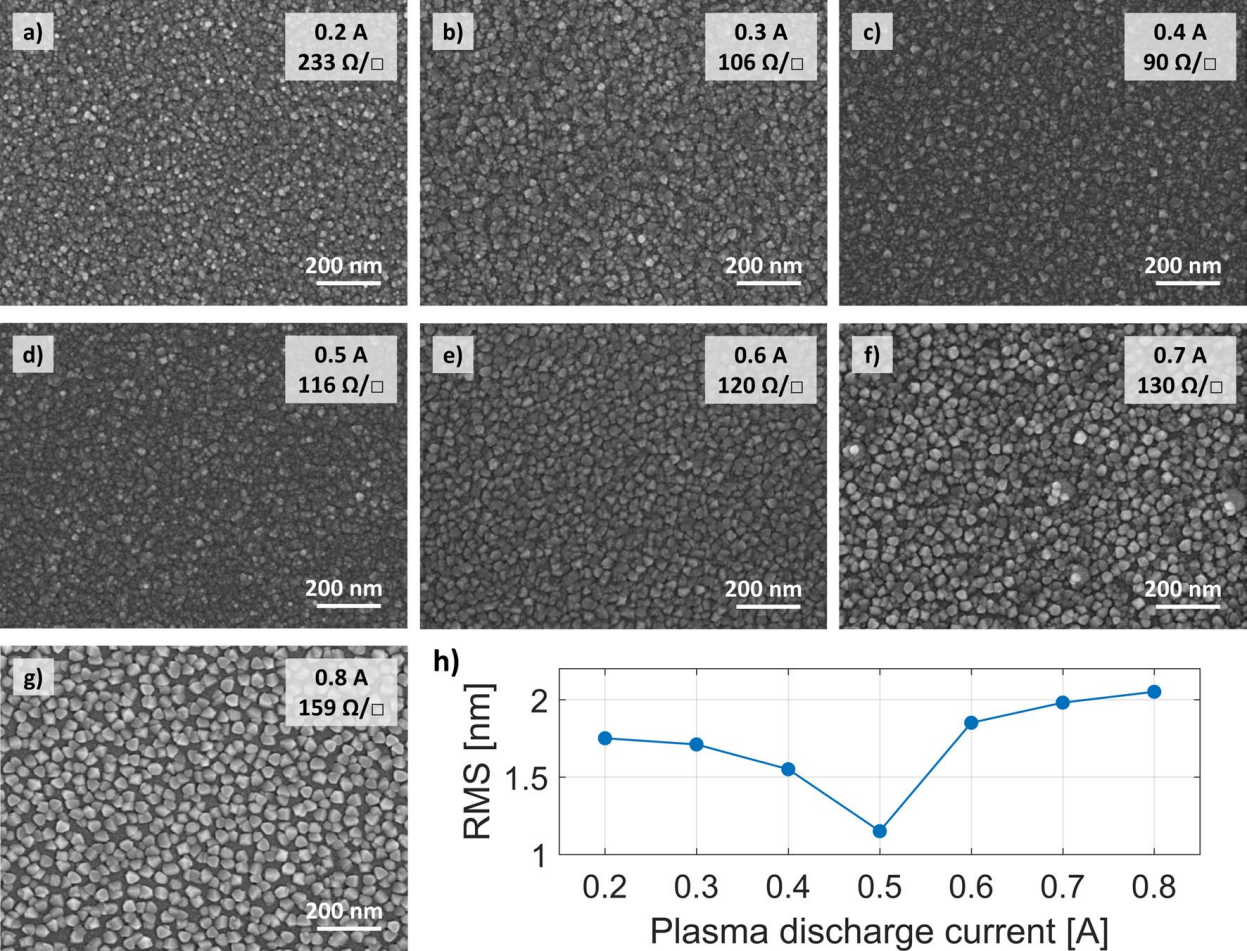
Surface morphology analysis of the ITO films deposited with oxygen ion assistance at different discharge currents and at a constant discharge voltage of 150 V. All samples are 70 nm thick and deposited at a rate of 10 Å/s. (**a–g**) SEM photographs. The discharge current used for each sample and the sheet resistance measured in the Hall system are highlighted in the upper right corner. (**h**) Surface roughness RMS from AFM scans of ITO 70 nm films deposited with oxygen ions assistance as a function of the discharge current. The data was gathered from a 3 µm × 3 µm area for each sample.

In the second series of samples deposited with the assistance of oxygen plasma we varied the discharge current between 0.2 A to 0.8 A, whereas the discharge voltage was held constant at 150 V. The voltage value was chosen to ensure stable plasma conditions over the widest possible discharge current range. This time the measured resistivity values exhibited a well-like shape trend (Fig. 5a). Although, for most of the samples the resistivity value oscillated around 6 × 10^–4^ Ωcm again, the samples deposited with the extreme plasma discharge currents presented different electrical characteristics, with much higher resistivity values. The measured carrier concentration and mobility dependencies (Fig. 5b) and acquired SEM images (Fig. 6) indicate that the resultant resistivity depends on the subtle interplay between the carrier characteristic parameters and morphology of the sample. The general trend is that with the increase of discharge current value more amount of oxygen gas is converted to plasma, which leads to a reduction of the number of oxygen vacancies (thus decreased concentration) and larger crystallites. Except for two extreme scenarios, the increase in carrier mobility seems to balance the effect of reduced electron concentration. However, at a discharge current of 0.2 A, the high density of grain boundaries additionally reduces the mobility of the carriers, while the additional drop in carrier concentration at high ion intensity of 0.8 A may indicate that the strong ion flux also affects the amount of Sn sites or causes some other structural changes.

The lowest sheet resistance of 90 Ω/□ was obtained for layers deposited with the discharge current of 0.4 A, the same as used in the previous series of plasma-assisted samples. The influence of the discharge current value on the surface morphology can also be detected in the RMS value measured with the AFM microscope (Fig. 6h). Initially, a decrease in surface roughness is observed as the crystallite size increases. However, above a certain ion flux threshold (discharge current of 0.5 A), the separation between the ITO clusters starts to become larger, and consequently, the RMS value starts to rise again.

**Figure 7:**
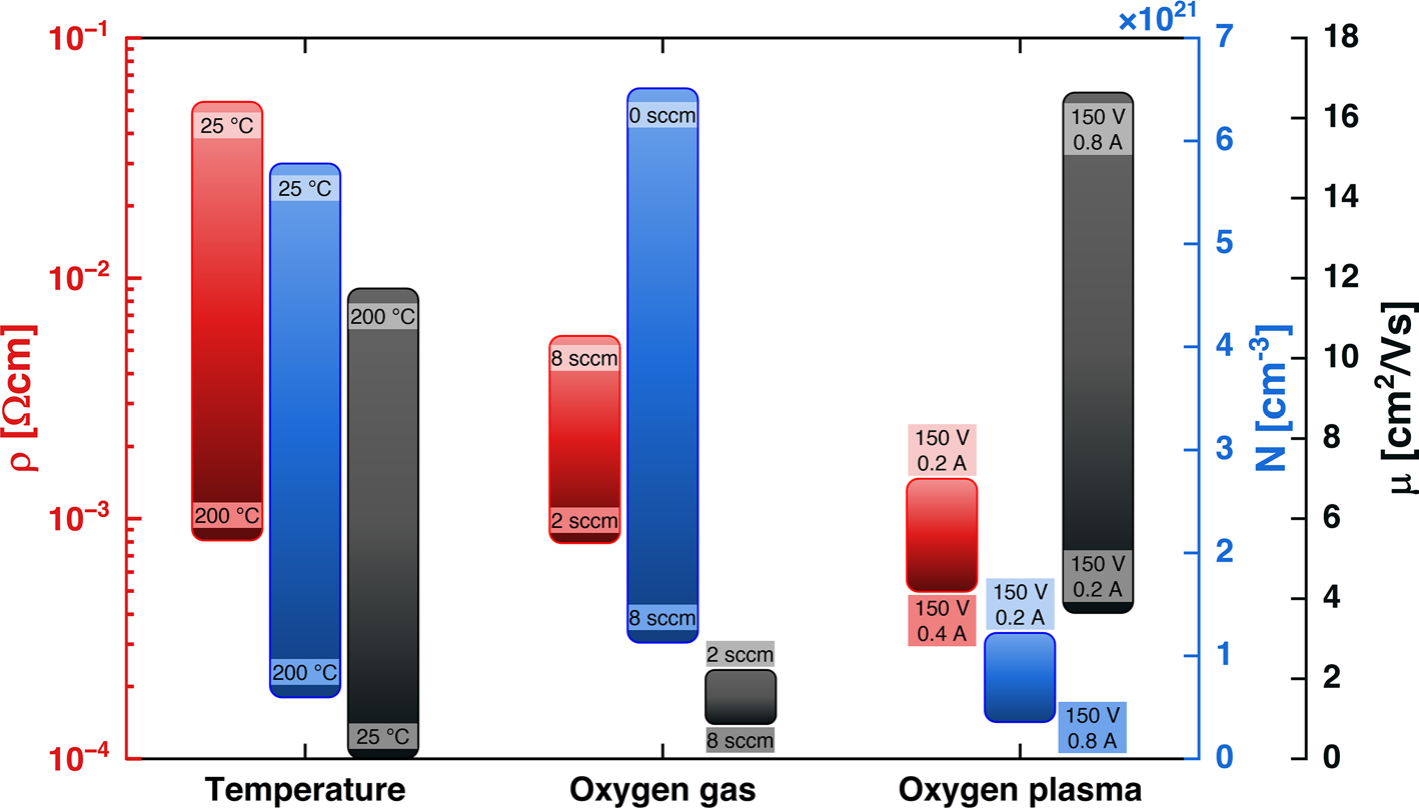
A comparative study of the electrical properties of 70 nm thick ITO films under varying deposition parameters. The color of every bar on the chart corresponds to a particular electrical property, with red representing resistivity, blue carrier concentration and black electron mobility. The graph is divided into three sections, each corresponding to the modification of different process parameters: substrate temperature (left), oxygen flow (center) and plasma discharge voltage and current (right). The length and position of each bar indicates the range of parameter values that can be achieved by varying process conditions. The labels on the bars specify the set of process parameters that resulted in ITO film with the highest or lowest value of each electrical property within each section. From the first group, it can be seen that one should increase the temperature to obtain low resistivity ITO films. However, this comes at the expense of low carrier concentration. Varying the oxygen gas flow (2nd section) helps to obtain samples with a broader range of carrier concentration values and moderate resistivity, although the mobility of carriers is highly limited. Finally, the use of oxygen plasma (3rd section) makes it possible to achieve the ITO film with the lowest resistivity and the highest carrier mobility, but within a limited concentration range.

To summarize the results presented so far, it can generally be said that the use of oxygen plasma seems to be obligatory to obtain high-quality ITO layers in the e-beam process with a substrate temperature of 80 °C and a deposition rate of 10 Å/s. The samples produced with the assistance of ions have the lowest resistivity, the smoothest surface, and at the same time, maintain a reasonable level of carrier mobility and concentration (Fig. 7). Both the adjustment of the plasma discharge voltage and the discharge current allow the properties of the ITO electron gas to be tuned, although the second approach permits this to be done over a wider range of values. Moreover, the use of oxygen plasma allows to omit the process of annealing, as our samples offer comparable or even better electrical properties than those subject to post-annealing treatment^2, 15, 35^.

## Optical properties of ITO films

**Figure 8:**
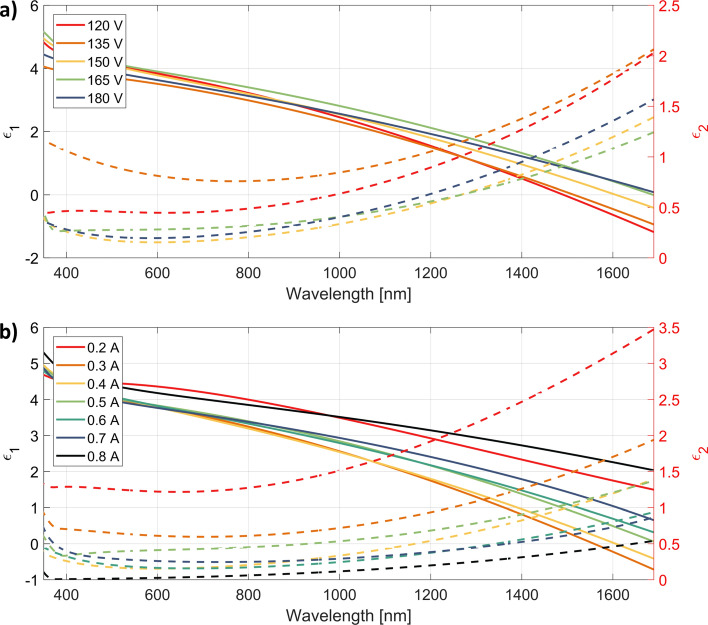
Electric permittivity dispersion recovered from the developed ellipsometric model for ITO samples deposited with varying (**a**) discharge voltage and (**b**) discharge current. A solid line corresponds to the real part of the permittivity, while the dotted one to its imaginary part.

Samples with the most promising electrical and morphological properties, i.e., those which were deposited in the presence of oxygen plasma, were selected for further characterization of the optical properties. Variable angle spectroscopic ellipsometry measurements performed over a wide spectral range have allowed us to reconstruct the permittivity dispersion curves of ITO films. The parameters of the oxygen ions flux have a significant effect on the optical constants, both for samples obtained at variable discharge voltage and at different discharge currents (Fig. 8). This is expected because the optical response of ITO layers in the NIR spectral range originates mainly from the properties of the ITO electron gas and, as we have shown in the previous section, both carrier concentration and mobility are affected by the oxygen plasma. The general observation is that higher discharge voltages and currents yield lower values of the imaginary part of the permittivity (Fig. 8a,b, respectively), which can be linked with the reduced carrier concentrations in these samples. At the same time, the values of the real part of the permittivity increase, which is consistent with the Kramers–Kronig relations, and indicate that the samples have become less metallic (see detailed discussion in the next section).

**Figure 9:**
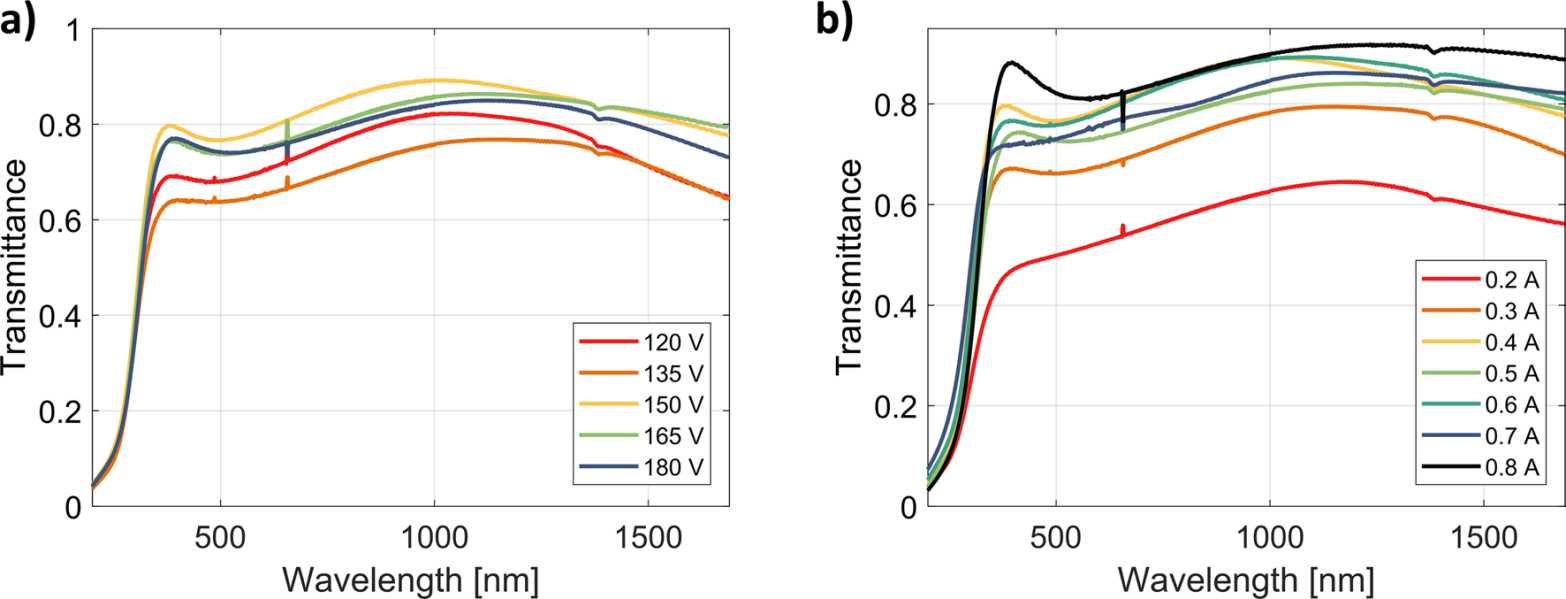
Comparison of the transmittance of ITO samples deposited with the presence of oxygen plasma as a function of (**a**) discharge voltage (with current set to 0.4 A) and (**b**) discharge current (with voltage set to 150 V). Peaks seen around 630 nm and 1400 nm wavelengths originate from the ellipsometer source instability in these regions.

The transmittance measurements, presented in Fig. 9, further confirm the correctness of the retrieved permittivity curves. The samples with the lowest values of the imaginary part of the dielectric constant are also the most transparent ones. In particular, the transmittance can be as high as 85% in the VIS/NIR range, depending on the choice of plasma parameters. The transmittance of the layer deposited at a plasma discharge current of 0.2 A is less than 60%, fundamentally different from the others in this series. The uniqueness of this sample was already recognized in the previous paragraph and is caused by insufficient ionization of the oxygen gas.

## Discussion

In this section, we have gathered the results from our electrical and optical measurements to highlight how the ENZ point of ITO layers can be tuned throughout the NIR spectral range, with the proper choice of the plasma discharge voltage and current. To provide cross-validation, we extracted the ENZ wavelength ${\lambda }_{ENZ}$, plasma frequency ${\omega }_{p}$ and the damping factor $\Gamma$ from both optical and electrical measurements. The values of these parameters were calculated using the following formulas:

2 $${\omega }_{p}=\sqrt{\frac{N{e}^{2}}{ {\varepsilon }_{\infty } {\varepsilon }_{0}{m}_{e}^{*}},}$$

3 $${\lambda }_{ENZ}=\frac{2\pi c}{\sqrt{{\omega }_{p}^{2}-{\Gamma }^{2}}},$$

where $c$ is the speed of light and $\Gamma$ is a damping factor obtained through relation $e/\mu {m}_{e}^{*}$. We paid particular attention to the value of the electron effective mass ${m}_{e}^{*}$. This parameter is typically considered as constant, however, we had to reassess this assumption due to the wide range of carrier concentrations in our samples. ITO conductance band is usually regarded as isotropic and parabolic, but it was found that the electron effective mass in this material is, in fact, a function of the free carrier density^54^. This accounts for the non-parabolicity of the conduction band and thus affects all other electrical and optical parameters.

The ENZ wavelength, plasma frequency and damping factor were calculated in three different ways. The first method relies solely on the results of the Hall measurements. In the second approach, we have calculated the values of λ_ENZ_, ω_p,_ and Γ directly from the Drude term in the ellipsometric model, taking into account the abovementioned dependence of ${m}_{e}^{*}$ on the carrier concentration. Finally, in the third procedure we read the λ_ENZ_ value directly from the real part of the permittivity curves (Fig. 8).

**Figure 10:**
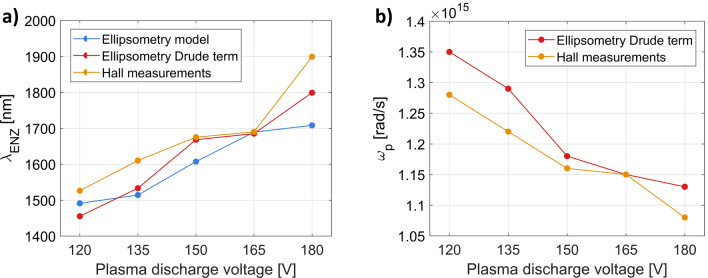
(**a**) λ_ENZ_ and (**b**) ω_p_ calculated with different methods, for the deposited 70 nm thick ITO layers as a function of the oxygen plasma discharge voltage. The plasma discharge current was set to 0.4 A and the oxygen flow was kept at 5 sccm. The temperature and rate of the deposition process were set to 80 °C and 10 Å/s, respectively.

In the case of the series of samples with varied plasma discharge voltage, λ_ENZ_ is almost a linear function of the voltage, as can be seen in Fig. 10a, while inverse proportionality is observed for the plasma frequency (Fig. 10b). All the presented curves are in good agreement, which demonstrates that the developed ellipsometry model accurately describes not only the optical, but also the electrical properties of the ITO samples. It also proves that the Drude term in the ellipsometric model is not affected by the presence of other oscillators and ENZ wavelength mainly depends on the plasma frequency. Finally, it is also worth noting that the control of the discharge voltage is a good tool to precisely design the operating spectral bandwidth of ENZ-based devices. The achieved range of λ_ENZ_ means in practice that the epsilon-near-zero properties can be tuned across S- to L-telecom bands (1460 to 2000 nm).

**Figure 11:**
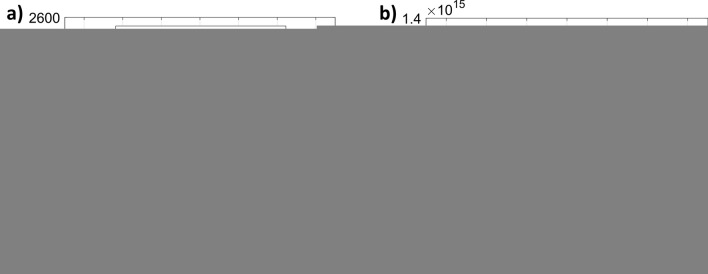
(**a**) λ_ENZ_ and (**b**) ω_p_ calculated with different methods, for the deposited 70 nm thick ITO layers as a function of the oxygen plasma discharge current. The plasma discharge voltage was set to 150 V and the oxygen flow was kept at 5 sccm. The temperature and rate of the deposition process were set to 80 °C and 10 Å/s, respectively.

Similar correlations between the optical and electrical measurements are observed for the second series of samples, when the discharge current was altered (Fig. 11). In the range between 0.3 A and 0.7 A, ENZ wavelength increases steadily from 1530 nm to around 1940 nm. The only points that stand out are 0.2 A and 0.8 A, where λ_ENZ_ shifts significantly towards the red, allowing ENZ wavelengths of 2400 nm to be reached. These two samples have similarly high resistivity, although, as indicated in earlier sections, the underlying reason for this behavior is different for each film. It is quite surprising that this deviation is not visible in the graph showing the plasma frequency (Fig. 11b). This observation yet again proves that the optical properties of ITO films, and in particular the ENZ wavelength, are very sensitive to the fabrication conditions.

## Summary

In conclusion, in this study, we present a holistic investigation of the electrical, optical, and morphological properties of 70 nm thick ITO films deposited by e-beam deposition under various process conditions. By means of Hall effect measurements, ellipsometry, AFM and SEM microscopes, we analyze the influence of deposition rate, substrate temperature, presence of oxygen gas and oxygen plasma on the quality of the ITO films. Based on our results, we propose a new procedure for the fabrication of high-quality ITO films, that does not require extensive substrate heating or post-annealing treatment, making the process compatible with polymers and thin metallic films. We discuss the possibility of tunning the carrier concentration and mobility and, therefore, the ENZ wavelength by controlling the discharge voltage and current of oxygen plasma. Finally, we show that for the 80 °C substrate temperature, it is possible to achieve ITO with resistivity as low as 5.2 × 10^–4^ Ω cm, smooth surface (RMS < 1 nm), high carrier concentration reaching 1.2 × 10^21^ cm^−3^ and high transmittance (85%) in the VIS/NIR spectrum. We anticipate that these findings will enable other researchers to successfully combine temperature-sensitive materials with ITO, which should allow them to take the next step in the construction of functional hybrid metal–semiconductor nanodevices.

## Methods

### Sample preparation

In our investigations, we used the electron beam physical vapor deposition (e-PVD) technique to develop a low-temperature and annealing-free fabrication procedure for ITO thin films with specific electrical and optical properties and a smooth surface morphology. The layers were evaporated on double-sided polished (RMS 0.3 nm) quartz glass with a diameter of 25 mm using a Lesker PVD75 system equipped with an additional plasma generating module. This substrate material was chosen primarily for its high optical transparency in the visible and near-infrared. But another motivation was that the amorphous SiO_2_ interlayer can be quite easily introduced by evaporation into more complex multilayer structures, hence making our conclusions about ITO fabrication conditions more versatile. Prior to the deposition process, the substrates were purged with nitrogen gas, then placed on the rotating plate and into the vacuum chamber of the PVD machine. The deposition process was started when the chamber was evacuated to a pressure of 5 × 10^–5^ Torr. The rotary plate was heated with two quartz lamps and its temperature was controlled with an accuracy of 0.1 °C. ITO films have been deposited at a rate in the range of 0.5 to 10 Å/s, with the pressure increasing to 2 × 10^–5^ Torr during the process at higher growth rates. For procedures where oxygen gas was used, the gas flow rate was kept constant at the desired level. The ITO layers were evaporated by heating pellets composed of 90 wt% In_2_O_3_ and 10 wt% SnO_2_. The thickness of the deposited films was set at 70 nm and its value was monitored during evaporation by a quartz crystal sensor. This is sufficient to observe different types of grain formation in the film but should also provide useful information on the kinetics of the initial stages of nucleation and growth.

The oxygen plasma generation system (End-Hall ion source) consisted of a tungsten wire and a discharge controller. High current (15–20 A) was passed through the wire, which in turn generated plasma by emitting electrons and ionizing the gas flowing through it. To achieve the desired plasma parameters the gas flow was adjusted and the discharge voltage and current were set to precise values. The discharge voltage translates into the energy of the oxygen ions, as a higher value accelerates the ions more strongly, while the discharge current describes the ionization level of the flowing gas. The latter parameter is coupled to an emission controller, which automatically adjusts the current passing through the tungsten wire to sufficiently ionize the flowing gas. In this way, the plasma parameters were effectively controlled by gas flow rate, discharge voltage and discharge current. For each set of parameters, we ran three independent deposition processes, each producing three samples. This allowed us to check the stability of the deposition conditions.

### Optical characterization

Variable angle spectroscopic ellipsometry measurements (RC2 from J. A. Woollam) were conducted under normal conditions to determine the deposited films’ complex optical constants, thickness, and roughness. The change in polarization state of the reflected light from the samples was measured at angles of incidence ranging from 60° to 75° by 5°, and described with ellipsometric parameters Ψ and Δ. A 3 M scattering tape was adhered to the backside of the sample to eliminate backside reflections. The created sample model consisted of a substrate and a single ITO layer with some roughness on its surface. The model was updated with the transmission intensity data to ensure a comprehensive analysis of the optical properties. To obtain the permittivity we use the general oscillator approach using a function consisting of three terms: (i) Drude, (ii) Tauc–Lorentz and (iii) Gaussian oscillators, typically used for this material^55, 56^.

### Morphological characterization

We employed a scanning electron microscope (SEM, Zeiss Sigma) and an atomic force microscope (AFM, Ntegra NT-MDT) to examine the surface morphology and determine the grain size of ITO films. We operated the AFM in semi-contact mode to evaluate the average root mean square (RMS) value of the surface roughness of the samples.

### Electrical characterization

Hall measurements in the Van der Pauw regime were carried out at room temperature (23 °C) using the Ecopia HMS-3000 system to determine the electrical properties. Electrical contact was obtained by soldering four tin contacts to the circular sample, evenly spaced around the edge. The applied magnetic field was 0.515 T and the current flowing through the sample was set at 100 µA.

## Data Availability

The data that support the findings of this study are available from the corresponding author upon reasonable request.
